# A Social Network Approach Reveals Associations between Mouse Social Dominance and Brain Gene Expression

**DOI:** 10.1371/journal.pone.0134509

**Published:** 2015-07-30

**Authors:** Nina So, Becca Franks, Sean Lim, James P. Curley

**Affiliations:** 1 Psychology Department, Columbia University, New York, NY 10027, United States of America; 2 Doctoral Program in Neurobiology and Behavior, Columbia University, New York, NY 10025, United States of America; 3 UBC Animal Welfare Program, 2357 Main Mall, Vancouver, BC, V6T 1Z4, Canada; Istituto Superiore di Sanità, ITALY

## Abstract

Modelling complex social behavior in the laboratory is challenging and requires analyses of dyadic interactions occurring over time in a physically and socially complex environment. In the current study, we approached the analyses of complex social interactions in group-housed male CD1 mice living in a large vivarium. Intensive observations of social interactions during a 3-week period indicated that male mice form a highly linear and steep dominance hierarchy that is maintained by fighting and chasing behaviors. Individual animals were classified as dominant, sub-dominant or subordinate according to their David’s Scores and I& SI ranking. Using a novel dynamic temporal Glicko rating method, we ascertained that the dominance hierarchy was stable across time. Using social network analyses, we characterized the behavior of individuals within 66 unique relationships in the social group. We identified two individual network metrics, Kleinberg’s Hub Centrality and Bonacich’s Power Centrality, as accurate predictors of individual dominance and power. Comparing across behaviors, we establish that agonistic, grooming and sniffing social networks possess their own distinctive characteristics in terms of density, average path length, reciprocity out-degree centralization and out-closeness centralization. Though grooming ties between individuals were largely independent of other social networks, sniffing relationships were highly predictive of the directionality of agonistic relationships. Individual variation in dominance status was associated with brain gene expression, with more dominant individuals having higher levels of corticotropin releasing factor mRNA in the medial and central nuclei of the amygdala and the medial preoptic area of the hypothalamus, as well as higher levels of hippocampal glucocorticoid receptor and brain-derived neurotrophic factor mRNA. This study demonstrates the potential and significance of combining complex social housing and intensive behavioral characterization of group-living animals with the utilization of novel statistical methods to further our understanding of the neurobiological basis of social behavior at the individual, relationship and group levels.

## Introduction

Study of social behavior in laboratory mice has primarily focused on short-term social encounters between familiar or unfamilar dyads [1,2]. The ancestors of laboratory mice are various substrains of Mus (*Mus musculus*, *Mus domesticus*, *Mus castaneus*, *Mus moloisha*) [3] and it is not possible to explicitly state what the natural ecology of a laboratory mice would have been. However, what all of these subspecies share in common is that they live in large groups with organized social structures and dominance hierarchies [4,5]. Attempts to explore social organization of mice have been limited, but do include studies of the spatial organization of wild mice, wild-derived mice and lab mice in semi-natural environments [6,7], and of the territoriality of male mice living in small groups in large arenas [8–10]. More recently, the spatial organization of small groups of mice in large arenas has been explored using automated tracking technologies [11–14]. However, these approaches have low resolution when distinguishing between highly similar behaviors that may occur rapidly or briefly (e.g. fighting, chasing), as well as those behaviors that occur between individuals whose identity may be obscured by other individuals or objects (e.g. nestboxes). Thus, these approaches may have significant limitations when used to describe complex social encounters in group-living environments.

Across species, understanding the social behavior and organization of any group of individuals requires evaluation of multiple levels of analysis: the individual, relationship, and group, and changes in these levels over time [15]. Social network analysis (SNA) has emerged an important methodological tool for unraveling social complexity at these multiple levels of analyses [16]. The advantage of SNA is that social groups can be analyzed by examining individual social behaviors within the context of their own direct relationships with others as well as their indirect relationships, thus building up a more complete picture of the social lives of group-living animals. SNA has most commonly been applied in behavioral ecology and primatology, for instance to explore the fitness benefits of individual differences in network position or how these are associated with variation in personality traits and genetic polymorphisms such as the serotonin transporter gene [15–19].

An important feature of living in a social group is that animals must quickly and flexibly exhibit dominance and subordinate behaviors according to the relative social position of their social partners. Social hierarchies in a group, once established, can be remarkably stable over time. In those studies of animals who are given the opportunity to spatially distribute themselves and to form social hierarchies, converging evidence from a diverse array of species including fish, primates and humans indicates the critical importance of the amygdala and hypothalamus in modulating this behavioral flexibility [20–27]. Although little is known as to the neurobiological basis of mouse social hierarchies living in large groups, studies of dyadic interactions have similarly implicated the medial and central nuclei of the amygdala [28–32] and the hypothalamus including the preoptic area [33–36] in the integration and evaluation of emotional and social information to eventuate rapid expression of contextually appropriate social behavior. Further, expression of the peptide hormone corticotrophin releasing factor (CRF) in these regions has also been shown to promote the learning and evaluation of social and emotional stimuli as well as dynamically modulating both dominant and subordinate behaviors dependent on social context in dyadic contests [37–39].

In this study, using trained observers, we collect precise, detailed information on the social behaviors (fighting, chasing, sniffing and allogrooming) occurring between individuals from a group of outbred CD1 mice living in a large three-dimensional vivarium who were tracked for 21 consecutive days and for over 100 hours in total. We use traditional dominance hierarchy statistical methodologies as well as novel approaches such as the Glicko pairwise-contest model to analyze social contest data and evaluate the spatial and temporal patterning of agonistic behaviors between individuals. We also utilize SNA to characterize the agonistic (fighting and chasing) as well as other social (sniffing and allogrooming) behavioral interactions of mice. Through comparison with other well-established methodologies of social dominance analysis, we propose that particular global and individual level network metrics are especially well suited to characterizing dominance power in animal social networks. We also test whether the social networks of different behaviors are related to determine if fighting and chasing are equally meaningful representations of dominance relationship status, to identify if other social behavior networks are related to agonistic network patterns and to understand what the functions of sniffing and allogrooming are in mouse social groups. We also analyze the relative gene expression of CRF of each individual in the medial and central nuclei of the amygdala, the medial preoptic area of the hypothalamus and also in the hippocampus as a control region. We hypothesized that those animals that were able to reach the top of dominance hierarchies would have higher relative expression in the amygdala and hypothalamus. Additionally, in the hippocampus, we investigate each individual’s relative gene expression of brain derived neurotrophic factor (BDNF). BDNF has been shown to be an important modulator of neural plasticity and social learning [40], and it is also elevated in mice who are quicker to learn their social dominance or subordinate status [41]. There is also evidence that hippocampal BDNF may specifically promote the ability of individuals to acquire knowledge about their social dominance [42,43]. We therefore hypothesized that more dominant animals would show increases in the relative expression of BDNF. Finally, we also test whether an individual’s social role was predictive of the relative expression of the glucocortiocoid receptor (GR) gene in the hippocampus.

## Materials and Methods

### Subjects and Housing

**Fig 1:**
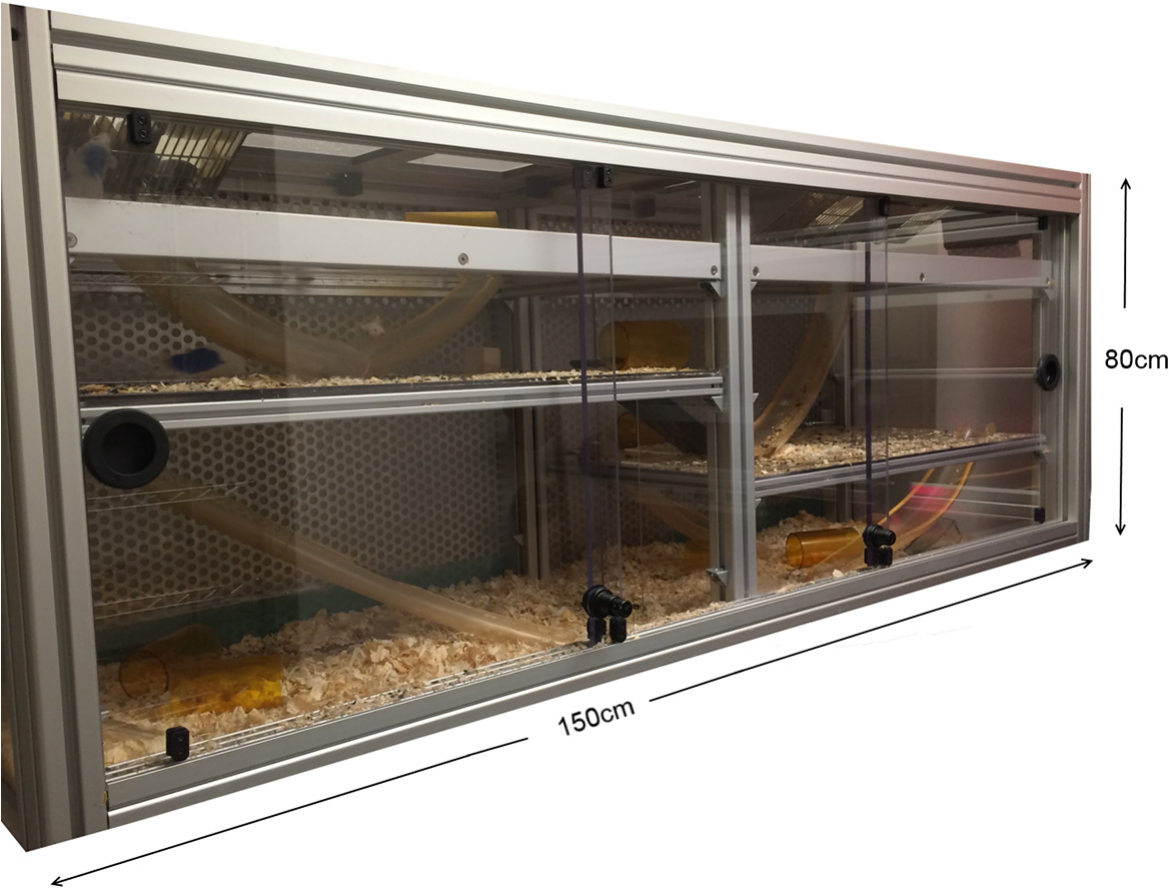
The mouse vivarium.

12 male outbred CD1 mice aged 6 weeks were purchased from Charles River and housed in groups of 4 for 3 weeks prior to testing. All mice were individually and uniquely marked by dying their fur with a blue, nontoxic, non-hazardous marker (Stoelting Co.). These marks remain for up to 12 weeks and only require one application, thus enabling each animal to be uniquely identified throughout the study. All animals were also given a unique and random two-letter code for identification purposes. At 9 weeks of age, all mice were weighed and placed into a large custom built mouse vivarium (length 150cm, height 80cm, width 80cm; Mid-Atlantic) **(Fig 1)**. The vivarium consisted of three sides of Plexiglas with sliding front doors and a metal backboard containing multiple holes for air circulation. The vivarium was split into two sides, each with two shelves. Standard food chow and water was provided ad libitum at the top shelf via cage lids that protruded through the vivarium roof. Animals could access each shelf via the backboard or via ramps and tunnels that connected each shelf and side. Multiple enrichment objects such as plastic igloos and wooden blocks were also provided. Floors of each shelf and side were covered with pine shaving bedding. The animals were kept in the same room throughout the study, with white lights (light cycle) coming on at 2400 hours and red lights (dark cycle) coming on at 1200 hours, at a constant temperature (21–24°C) and humidity (30–50%). Mice were housed in the Department of Psychology at Columbia University. All procedures were conducted with approval from the Columbia University Institutional Animal Care and Use Committee (ICAUC).

### Behavioral Observations

Starting the day after animals were placed into the vivarium and for 21 consecutive days, live behavioral sampling was conducted every ten minutes for between one and seven hours per day. At each time interval, trained observers recorded the location, behavior and social partner (target individual) of every mouse (focal individual) in the vivarium (see ethogram in **S1 Table**). On a typical day, observations began at 10 AM and ended at 5 PM, thus our sampling period covered the last two hours of the white light cycle and the first half of the red light cycle. All data were inputted live to an android device and uploaded to our secure server using Qualtrics (Qualtrics, Provo, UT). Fighting, chasing, allogrooming and sniffing data were used for all subsequent analyses.

### Data Structure & Matrix Organization

All recorded behaviors directed from one individual to another were organized into separate square matrices by each behavior (fighting, chasing, sniffing, grooming). These matrices are referred to as interaction frequency sociomatrices. In social network analysis they are also referred to as the valued data. Each row and column refers to a specific individual, with the initiator/giver of each behavior being represented by the row and the receiver of each behavior being represented in the column. As individuals cannot initiate behaviors against themselves no data exists in the diagonal of each matrix. Additionally, a fifth matrix was comprised (‘all aggression’ matrix) which is the sum of the entries for the fighting and chasing interaction frequency sociomatrices. These data are also referred to as directed or asymmetric data as individuals may direct behaviors more frequently to individuals than they receive those same behaviors from the same individuals. For other analyses, each interaction frequency sociomatrices are binarized. One set of binary matrices produced are the presence/absence binary matrices. Here, each cell of the binary matrix is given a ‘0’ if a ‘0’ exists in the corresponding cell in the frequency interaction sociomatrix. Cells are given a ‘1’ if any value greater than ‘0’ exists in the corresponding cell in the frequency interaction sociomatrix. These matrices capture whether ‘individual i’ was ever observed directed each behavior to ‘individual j’ in the matrix (a ‘1’ indicating the presence of that behavior and a ‘0’ indicating the absence of that behavior). A second binarizing method produces win/loss matrices. This method is only applied to the fighting, chasing and ‘all aggression’ behavioral matrices. Here, if ‘individual i’ directs a greater number of a particular behavior towards ‘individual j’ than it receives from ‘individual j’ then a ‘1’ is entered into the binary matrix in the cell [row i, column j] and a ‘0’ is entered into the binary matrix in the corresponding cell [row j, column i]. In this sense, ‘individual i' is considered the ‘winner’ and ‘individual j’ the ‘loser’. If individuals direct the same amount of behavior towards each other (i.e. they tie and there is no clear winner) then both cells get a ‘0’ [44,45]. This is performed for every potential relationship in the matrix. Both the presence/absence and win/loss binary matrices are considered directed, asymmetric data and are also referred to as adjacency matrices for the social network analysis. These operations are performed using the R package compete v0.1 [46].

### Dominance Hierarchy Analysis

Data analysis was undertaken primarily in R version 3.2.0 [47]. The following three global measures of dominance hierarchies were undertaken independently upon the fighting, chasing and all aggression interaction frequency sociomatrices. **a) Linearity**–The hierarchy linearity was assess using De Vries’ improved linearity index that corrects for unknown relationships [48]. This method computes a modified Landau’s h’ linearity index value which ranges from 0 (no linearity) to 1 (strictly linear). The probabilistic significance of this linearity was determined by performing 10,000 two-step randomizations whereby data within each matrix are permuted producing a range of h’ values against which the computed h’ value can be compared. This was calculated using the R package compete v0.1 [46]. **b) Steepness–**The steepness of a hierarchy is a measure of how large the absolute differences in dominance contest winning ability are between adjacently ranked individuals [49]. A steepness of ‘0’ would represent these differences being very small and a steepness of ‘1’ represents these differences being very large. Here, we calculate steepness using the R package Steepness v0.2.2 [50]. Briefly, a dyadic dominance index (Dij) is calculated which is equal to the proportion of wins and losses corrected for the frequency of interactions. Dij then can be used to calculate normalized David’s Scores (DS) for each individual (see below for more information). Regressing the normalized DS against the individual rank of each DS determines the steepness. The probabilistic significance of hierarchy steepness (i.e. against the null hypothesis of random win likelihoods for individuals across all relationships) is then computed by performing 10,000 randomizations. We also report the steepness calculated based on the Pij dominance indices which do not account for individual differences in frequency of interaction and are simply a ratio of total wins to total interactions. **c) Directional Consistency (DC)–**The DC index is calculated for each sociomatrix and ranges from 0 (where there is equal likelihood that directed behavior occurs in each direction between individuals) to 1 (where all directed behavior is always in the most frequent direction) [51]. DC is calculated across all dyads and is equal to (H-L)/(H+L) where H is the number of times the behavior occurred in the most frequent direction and L is the number of times the behavior occurred in the least frequent direction within each dyad. The DC is a commonly used and straightforward index to assess dominance structure but there is no significance testing associated with it. We calculated DC using the R package compete v0.1 [46].

### Individual Dominance Measures

As all agonistic social hierarchies were found to be significantly linear according to the modified Landau’s h’ linearity index, we sought to determine the ranking of each individual. **a) Inconsistencies and Strength of Inconsistencies (I&SI) ranking–**The most commonly used individual ranking method is the I&SI ranking [52]. This method seeks to reorder the rows and columns of each win/loss matrix such that more dominant individuals are placed in earlier rows and columns. In a perfectly ranked and ordered matrix, all ‘1’s would be above the diagonal and all ‘0’s would be beneath the diagonal. As this is rarely achieved, this method computes those matrices that minimize the number of rank inconsistencies, i.e. those matrices that possess the fewest ‘1’s under the diagonal. The final rank order is equivalent to the row/column order of individuals. This method does not always produce one optimal solution, for a given matrix there may be several solutions. For those matrices where more than one ranking solution was found, we calculated the average I&SI rank across all optimal solutions found for each individual. We calculated I&SI ranks using the R package compete v0.1 [46]. To calculate each individual's I&SI rank in our data we determined all potential rank order solutions for each behavior matrix and averaged the ranks for each individual to determine their final average I&SI rank. **b) David’s Scores–**We calculated the David’s score of each individual, which is a cardinal score of the overall success of an individual at winning contests relative to the success of its opponents [53–55]. This is achieved by calculating the dyadic proportion of wins (Pij) for each individual i in contests with another individual j. If individuals never interact, then Pij and Pji = 0. The DS of each individual is then calculated by adjusting for the raw versus weighted sums of Pij for i and j. David’s Scores were calculated using the R package Steepness v0.2.2 [50].

### Glicko Rating System

The Glicko Rating System is a dynamic paired comparison model that calculates a 'rating' for each individual based on the temporal sequence of wins and losses of each individual [56]. In brief, individuals that win contests gain rating points and individuals that lose contests lose ratings points. The number of ratings points gained or lost is dependent upon the difference in ratings between those two individuals prior to the contest. For instance, individuals that are rated much more highly than their opponent will gain relatively fewer ratings points than individuals who beat opponents that are rated similarly or higher than themselves. Likewise, individuals who lose to higher rated opponents will only lose relatively few ratings points, but individuals that lose to lowly rated opponents will lose far more ratings points. The Glicko system was developed by Mark Glickman as an improvement to the well-known Elo ratings system that is commonly used for assessing rankings of a group of individuals that compete against each other in paired contests [57]. There are two major advantages of the Glicko rating system over the Elo rating system. Firstly, in addition to computing a rating for every individual after every event period, the Glicko system also calculates a 'ratings standard deviation'. Larger standard deviations reflect greater uncertainty in the 'real' rating of an individual, whereas smaller standard deviations reflect greater certainty in that rating. The more contests an individual takes part in, the smaller the rating standard deviation becomes and the more certain the rating. The second major advantage of the Glicko system is that individuals who do not occur very frequently in contests will have increases in their rating standard deviations, reflecting an increased uncertainty in their real ratings with the passage of time. Further, with the Glicko system, the number of ratings points gained by each individual in a paired contest is not governed solely by the ratings difference between the two individuals but also by each individuals' ratings deviation. This can lead to individuals gaining and losing unequal ratings points from the same contest. The Glicko ratings algorithm contains one positive constant 'c' that governs the size of change in ratings deviations over time. The value of 'c' is user-defined, with higher values of 'c' leading to increased average ratings deviations per individual. Following the guidelines of Glickman (1999) [56], for our analyses we have chosen a value of 'c' of 1, but we also demonstrate that the value of 'c' chosen is relatively trivial for our data as our results are robust over a range of 'c' values (**S1 Fig**). The Glicko analysis was performed using the PlayerRatings package v1.0 in R [58]. Data was structured in the temporal order of pairwise contests. The Glicko analysis was performed on data containing fights only, chases only and all aggressive behaviors (fights and chases combined). Glicko Ratings were analyzed using the ‘PlayerRatings’ package in R. Intercorrelations between individual dominance ratings and ranks were analyzed using Spearman rank tests in R.

### Social Network Analysis

For social network analysis, we converted interaction frequency sociomatrices for each behavior (fighting, chasing, sniffing, grooming) into presence/absence binary adjacency matrices (see Data Structure and Matrix Organization section above). Network measures were analyzed using the ‘igraph v0.7.1’ and ‘sna v2.3–2’ packages in R, and UCINET 6 [59–61]. Networks were visualized using Gephi v0.8.2 using the GC-Viz plugin layout algorithm [62]. Given the vast array of potential social network metrics that could be analyzed, we chose a small subset that met the following criteria: i) those that are appropriate for asymmetric directed networks–i.e. not those that require matrices to be symmetrized, ii) those that make theoretical sense in the context of this study–i.e. those that are informative to describing the hierarchical nature of the social group and individual differences in influence and power.

#### Global Network Measures

To compare global network structural similarities and differences across behavioral networks we applied three common measures of inter-connectivity [63]: a) ***Density***–the proportion of all possible ties that exist in the network; b) ***Average Path Length***–the average number of steps between any two individuals in the network. Unreachable nodes are given the maximum path length; c) ***Reciprocity***–the proportion of mutual ties that exist in the network. We also analyzed the degree to which power is unequally shared within each network. d) ***Out-degree Centralization***–This measures the degree of variability in the distribution of out-degree across all individuals in the network normalized to the maximum achievable centralization for a network of the same size (which occurs when one individual is at the center of a star network graph and is the only individual to connect to any other individual) [63]. Higher scores reflect greater variability in the distribution of out-degree between individuals, indicating that the power of individuals varies substantially. e) ***Out-closeness Centralization***–Individuals with high out-closeness centrality are highly connected to many individuals in short steps. The global network centralization measure reflects how variable the distribution of these centrality scores are across individuals in the network normalized to a theoretical maximum achieved with a star network graph [63]. Networks with high out-closeness centralization scores possess individuals with highly unequally distributed power.

#### Individual Network Measures

To compare the position of individuals in each behavioral network, we computed the following measures of centrality and influence for each presence/absence binary matrix [63]. **a) *Out-degree & In-degree*–**The number of ties of each individual to (out) and from (in) all other individuals. **b) *Out-closeness & In-closeness Centrality*–**Whereas ‘degree’ measures only take into account the total number of ties of each individual, the closeness centrality measures reflect how closely tied each individual is to every other individual in the network. High out-closeness centrality indicates an individual is closely connected to many individuals in relatively few steps; high in-closeness centrality is reflective of having many other individuals being closely connected to an individual via relatively short paths. **c) *Betweenness centrality***–This measure indicates how proportionally frequently an individual lies on shortest paths between all other pairs of individuals in the network. We further examined two measures of individual power by analyzing the win/loss binary matrices for fighting, chasing and all aggression. **d) *Bonacich’s Power Centrality*** *–*This measure defines an individual’s power by the sum of the power of those individuals to which they are tied [64]. The nature of this power is further defined by a power exponent (β). When negative, this results in powerful individuals being those who have directed ties to many other individuals who do not have very many outgoing directed ties themselves. In this sense, individuals become more powerful as individuals that they are tied to become weaker. The minimum value of β should be [1 / (the maximum out-degree of any individual)] [64]. Here, the β value used is -0.09. **e) *Hub Centrality*** *–*This measure was developed by Kleinberg [65] to evaluate the connectivity of webpages. Briefly, individuals that have a high number of outgoing ties will have higher hub scores especially if these outgoing ties are directed to ‘authorities’–these are individuals who receive a lot of ties from other individuals.

#### Social Network Statistical Analysis

Statistical analysis of global metrics were undertaken using bootstrapping methods provided by UCINET [59]. We tested whether the density of each network was significantly different from a theoretically maximally connected network of density = 1 by randomly permuting ties within each network 5,000 times to generate a distribution of network densities to which the observed value can be compared. Comparison of densities between behavioral networks was performed using a bootstrapped version of the paired t-test with 5,000 permutations in UCINET. We also tested whether the patterning of relationships between individuals was similar or dissimilar across different behavioral networks by testing whether they were significantly associated with each other using the quadratic assignment procedure (QAP) regression in UCINET [59,66] which accounts for non-independence in the data. The QAP is similar to a Mantel test. A standard regression is computed across all corresponding cells for each pair of behavior networks to be tested. Then the cells of one matrix are randomly permuted and the regression is repeated. This permutation procedure is repeated 5,000 times, following which the observed regression coefficient is compared to the distribution of coefficients to determine a p-value. We also tested whether the fighting and chasing networks had a hierarchical like structure by testing whether the maximum out-degree of each network significantly differed from random by computing the out-degree for 5,000 random networks drawn from a Bernoulli graph distribution that had the exact same number of nodes and graph density as each behavior network [67]. Similarly, we tested whether the grooming network had significantly higher reciprocity than expected by chance by comparing to 5,000 random networks with the same number of nodes and graph density. P-values were determined by calculating the proportion of computed maximum out-degrees that were greater than or equal to the observed value for each network. This was performed using the SNA package in R [60]. Individual variation in network metrics were compared across behavior networks using Spearman rank correlations in R. As values for the fighting network were not normally distributed, this non-parametric approach was taken for all tests (even those not involving the fighting network) to be consistent and conservative across tests. As each measure (e.g. sniffing out-degree) could be correlated with three other behavior networks (fighting, chasing, grooming), we used a Bonferonni corrected significance level of p = (0.05/3) 0.0167. Intercorrelations between individual network metrics and dominance ranks were also analyzed using Spearman rank tests in R.

### Real Time PCR analysis

Immediately following the last behavioral observation, mice were euthanized by cervical dislocation and brains rapidly removed and paced into hexane cooled by dry ice. Brains were stored in a -80°C freezer until dissection. Samples of the medial amygdala (MeA), central amygdala (CeA), medial preoptic area of the hypothalamus (mPOA), and whole hippocampus (ventral and dorsal) were collected using Harris Micro-Punches with reference to coronal cross-sections from the Mouse Brain Atlas [68]. The MeA and CeA were each collected bilaterally in 1mm diameter punches from Bregma -0.82mm to -1.46mm. The mPOA was taken as one 1mm diameter area along the midline from Bregma +0.14mm to -0.7mm. The hippocampus was collected bilaterally from Bregma -0.82mm to -1.46mm by extracting tissue within the boundaries of the hippocampal structure using a 0.5mm diameter Micro-Punch. RNA was isolated from the dissected brain regions of each male using the AllPrep DNA/RNA Mini Kit (Qiagen) and reverse transcribed to cDNA using the SuperScript III First-Strand Synthesis System for RT-PCR applications (Invitrogen). Quantitative RT-PCR was performed with 1 μL of cDNA using an ABI 7500 Fast Thermal Cycler and the Fast SYBR Green Master Mix reagent (Applied Biosystems). All primer probes (Sigma-Aldrich) were designed to span exon boundaries ensuring amplification of only mRNA. For each gene, C_T_ values were normalized to cyclophillin A (endogenous control). Relative expression values were obtained by the ΔΔC_T_ method with fold-change being determined respective to the individual with the lowest expression value for each gene in each brain region. The following validated quantitative PCR primers were used for mRNA analysis [69]: brain-derived neurotrophic factor—BDNF (Forward: CCATAAAGGACGCGGACTTGTACA, Reverse: AGACATGTTTGCGGCATCCAG); Ccorticotrophin-releasing hormone—CRF (Forward: GGGAAGTCTTGGAAATGGC, Reverse: GCAACATTTCATTTCCCGAT); cyclophilin A—CYPHA (Forward: GAGCTGTTTGCAGACAAAGTTC, Reverse: CCCTGGCACATGAATCCTGG); glucocorticoid receptor—NR3C1 (Forward: AACTGGAATAGGTGCCAAGG, Reverse: GAGGAGAACTCACATCTGGT). Intercorrelations between relative gene expression and individual network metrics and dominance ranks were analyzed using Spearman rank tests in R. We also conducted separate principal components analyses (PCA) for the fighting and chasing networks on individual network metrics and dominance ranks used in the correlation analysis. This was done using the FactoMineR R package v1.30 [70]. Each produced one component ‘dominance’ that accounted for the majority of variance (87%) and thus individual components scores were also correlated against the expression of each gene. Note—one sample of mPOA CRF was contaminated and disregarded from analyses.

## Results

### 1. Social Dominance

**Fig 2:**
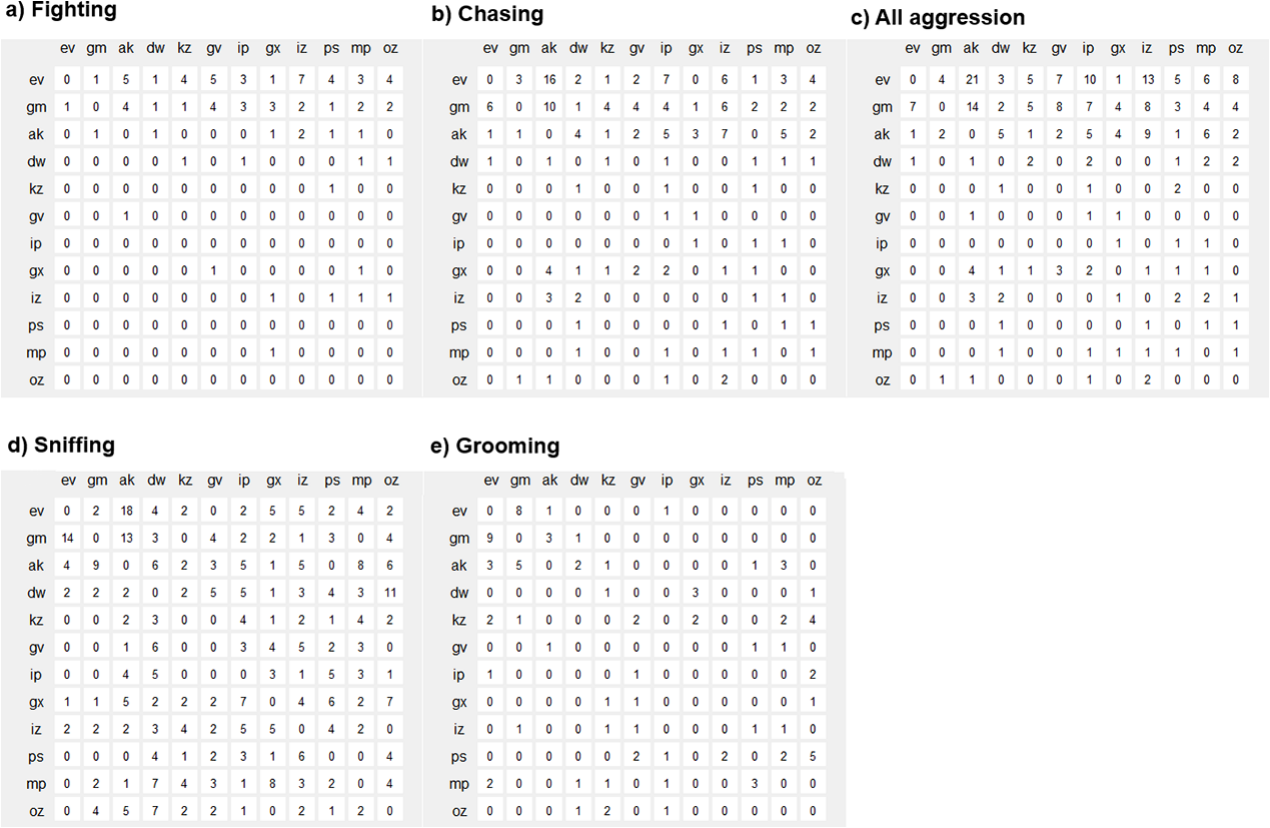
Frequency of observed social interactions. a) Fighting, b) Chasing, c) All aggression, d) Sniffing, e) Grooming. Behaviors are directed from individuals in rows to those in columns.

The frequency interaction sociomatrices and binarized sociomatrices are presented in **Fig 2** and **S2 and S3 Figs.**

#### Dominance Hierarchy analysis

All three agonistic interaction frequency sociomatrices (chasing, fighting, all aggression) possessed significant linearity of social dominance (**Table 1**). The observed values of Landau's modified h' value from each agonistic interaction frequency sociomatrix was significantly higher than the distribution of possible h' values generated from permuting each matrix 10,000 times (**S4 Fig**). Further, the directional consistency of the fighting sociomatrix was extremely high with 93% of all fights occurring in the direction of more dominant to more subordinate individual (**Table 1**). The directional consistency of the chasing sociomatrix was also very high, with 71% of all chases occurring in the direction of more dominant individual to more subordinate individual. A third complementary measure of the structure of social hierarchy is the steepness of the hierarchy. This is a measure of how much inter-individual variability exists in the overall win success of contests. We found that when considering fighting and chasing matrices separately or when considering all aggressive acts together that all hierarchies had highly significant steepness (p<0.0003; **Table 1**)

**Table 1:** Summary statistics of dominance hierarchy analysis for chasing, fighting and all aggression. (Modified h' = Landau’s modified h’ index of linearity; p(Modified h’) = p-value for Landau’s modified h’ index of linearity after 10,000 randomizations; DC = directional consistency; Pij steepness = Pij index of steepness of hierarchy; Dij steepness = Dij index of steepness of hierarchy which controls for frequency of interaction; p(Dij steepness) = p-value of Dij after 10,000 randomizations.

	Fighting	Chasing	All aggression
**Modified h’**	0.618	0.679	0.711
**p(Modified h’)**	0.001	0.000	0.000
**DC**	0.927	0.711	0.750
**Pij steepness**	0.379	0.575	0.596
**Dij steepness**	0.212	0.334	0.421
**p(Dij steepness)**	0.0038	0.0001	0.0001

For each behavior matrix, we also calculated individual measures of dominance—David's Scores and the I&SI rank order of individuals (see Methods). It should also be noted that the body weights of individuals do not predict any measure of dominance rank (all p >0.2 Spearman’s Rank test). Further, the location of attack did not affect the likelihood of winning or losing by any individual as has been observed in previous studies [71].

#### Temporal analysis

**Fig 3:**
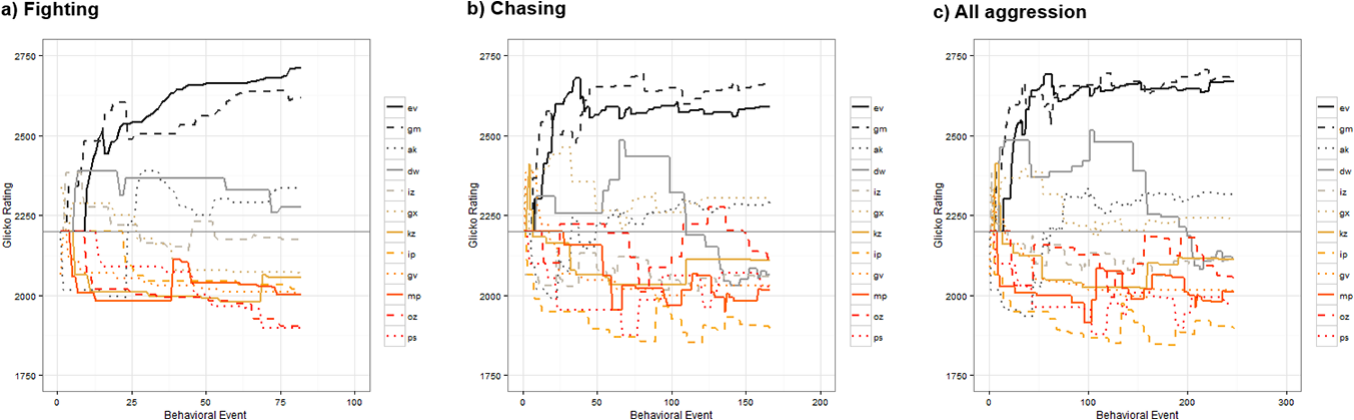
Temporal changes in individual Glicko Dominance Ratings. a) Fighting, b) Chasing, c) All aggression. All individuals arbitrarily begin with a 2200 rating and ratings are recalculated for each individual following a contest.

The results of the Glicko analysis on the pairwise fighting, chasing and all aggressive behavior data are presented in **Fig 3**. The final Glicko ratings and rankings of each individual are shown in **Table 2**. With the fighting, chasing and all aggression data, four individuals finished with a rating above their initial starting rating of 2200. As can be seen from **Fig 3**, the consistency of the rank order of Glicko ratings was very high across time with very little change-over in rankings of individual animals occurring after the initial first third of the data. This was especially the case for the temporal distribution of individual differences in fighting ratings. Further, as shown in **S1 Fig** the general pattern of individual differences in the distribution of Glicko ratings was very consistent over a range of 'c' constant values (1–20). As can also be seen from **S5 Fig**, there exists incredibly high consistency in individual ranks of final Glicko ratings for fighting across this range of c-values, therefore making the choice of 'c' value for our data relatively trivial.

**Table 2:** Individual measures of social dominance for A) Fighting, B) Chasing and C) All Aggression sociomatrices.

**A**					
**Individual**	**DS**	**Glicko rating**	**IS&I rank**	**DS rank**	**Glicko rank**
**ev**	23.6	2711	1.2	1	1
**gm**	19.8	2617	1.7	2	2
**ak**	3.3	2337	3.8	3	3
**dw**	0.2	2276	7.1	4	4
**kz**	-3.2	2057	8.2	5	7
**gv**	-3.7	2008	4.1	6	9
**ip**	-4.9	2014	9.1	8	8
**gx**	-6.5	2066	5.2	9	6
**iz**	-3.8	2175	5.6	7	5
**ps**	-8.2	1898	10.9	10	12
**mp**	-9.7	2003	9.8	12	10
**oz**	-7.0	1903	11.2	11	11
**B**					
**Individual**	**DS**	**Glicko rating**	**IS&I rank**	**DS rank**	**Glicko rank**
**ev**	25.2	2590	2.4	2	2
**gm**	32.1	2659	1.0	1	1
**ak**	9.3	2293	4.0	3	4
**dw**	-6.4	2066	8.4	7	7
**kz**	-3.4	2108	6.4	5	6
**gv**	-5.1	2032	6.3	6	9
**ip**	-20.1	1898	11.3	12	12
**gx**	8.1	2305	2.6	4	3
**iz**	-9.7	2060	8.6	9	8
**ps**	-11.7	2028	11.4	11	10
**mp**	-9.9	2016	5.9	10	11
**oz**	-8.5	2112	9.9	8	5
**C**					
**Individual**	**DS**	**Glicko rating**	**IS&I rank**	**DS rank**	**Glicko rank**
**ev**	36.7	2669	2.0	2	2
**gm**	39.0	2686	1.0	1	1
**ak**	11.3	2326	3.3	3	3
**dw**	-5.7	2112	7.0	7	5.5
**kz**	-6.5	2112	9.7	8	5.5
**gv**	-5.2	2014	5.0	6	9
**ip**	-23.1	1895	11.0	12	12
**gx**	3.6	2238	3.7	4	4
**iz**	-4.4	2109	6.0	5	7
**ps**	-18.6	1973	12.0	11	11
**mp**	-13.5	2012	8.0	10	10
**oz**	-13.5	2049	9.3	9	8

(DS = David’s Score; Glicko rating = final rating calculated by Glicko rating system; I&SI rank = rank calculated using I&SI algorithm; DS rank = rank calculated using David’s Scores; Glicko rank = rank calculated using final Glicko rating).

#### Consistency of ranking methods

We examined the consistency between ranking methods based on cardinal values (David's scores), minimizing matrix inconsistencies (I&SI) and temporal pairwise contests (Glicko). All three methods were extremely significantly positively correlated to one another using Spearman rank correlation tests even after correcting for multiple comparisons (**Table 3**, Spearman's rho range between 0.73–0.96, all p < .0001). Thus, the ranking of individuals within each behavioral context was extremely similar regardless of the ranking method chosen. When comparing the consistency of individual competitive ability between fighting versus chasing behaviors, dominance scores were significantly correlated for I&SI ranks (rho = 0.80, p<0.005), Glicko scores (rho = 0.69, p<0.05), and David's scores (rho = 0.77, p<0.005).

**Table 3:** Individual consistency between ranking methods.

	Fighting rho	Fighting-p	Chasing rho	Chasing p	All aggression rho	All aggression p
**I&SI–DS**	0.83	<0.005	0.87	<0.001	0.96	<0.0001
**I&SI—Glicko**	0.86	<0.001	0.73	0.01	0.84	<0.001
**DS—Glicko**	0.89	<0.0001	0.92	<0.0001	0.92	<0.0001

(DS = David's scores)

### 2. Social Network Analysis

**Fig 4:**
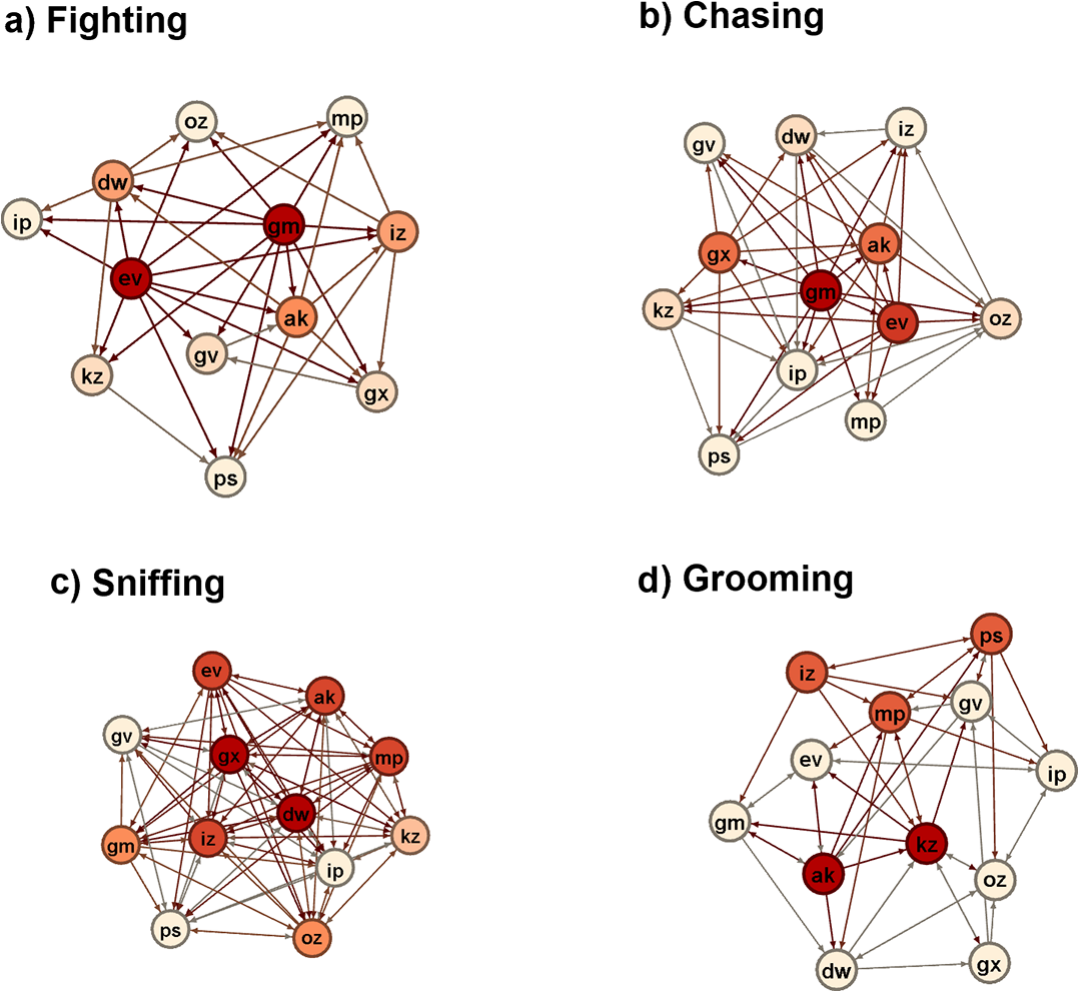
Visual representations of the a) fighting, b) chasing, c) sniffing and d) grooming social networks. Nodes are colored from cream to red based on out-degree. More red colors represent individuals with a relatively higher out-degree. Fighting and chasing networks are based on the win-loss binary matrices. Sniffing and grooming networks are based on the presence-absence binary matrices.

Using social network analysis, we sought to address three key questions: i) How similar or different are the fighting, chasing, sniffing and grooming networks to each other in their global structure? ii) Are the positions of individuals in one social network similar or different to their position in other behavioral networks? iii) Can an individual’s social dominance be characterized by individual differences in the fighting and chasing networks? Depending upon the metric being evaluated, frequency interaction sociomatrices and/or binarized matrices were used (see Methods). These networks are visually represented in **Fig 4**.

#### i) Assessment of Global Network Structure

The binarized presence/absence networks were analyzed for group structure. Densities of networks varied from 31.1% of all potential ties between individuals being present in the fighting network to 82.6% of all potential ties being present in the sniffing network (**Table 4**). Using a bootstrap test with 5,000 samples, all binarized networks deviated significantly from a theoretical density of 1 (i.e. the condition in which all individuals would be connected). z-values ranged between -6.55 and -9.45 with p<0.0002 for all networks except for the sniffing presence/absence binarized network where z = -2.25, p = 0.031. Thus, all social networks possess significant social organization (see **S2 Table**). Significant dissimilarities between the densities of different networks were assessed using permutation paired-samples t-tests with 5,000 permutations to generate bootstrapped t-statistics and p-values. The density of the sniffing network was significantly larger than those of all other networks (all t-statistics > 4.39, all p-values < 0.001). The fighting (t = 3.21, p = 0.024) and grooming (t = 1.70, p = 0.042) networks were also significantly less dense than the chasing network. There was no significant difference in the density of the grooming and fighting networks. Similar to the density measures, the fighting network has a very high average path length meaning that individuals are not heavily interconnected, whereas the sniffing network has a low average path length indicating high interconnectivity. Consistent with the other measures, the sniffing network had the highest reciprocity indicating more balanced interactions. The fighting network had the lowest reciprocity indicating very unbalanced relationships. Of note, the grooming network’s reciprocity showed a trend (p = 0.062) to be significantly higher than expected by chance compared to random networks of the same size and density.

**Table 4:** Group level network metrics for the fighting, chasing, sniffing and grooming presence/ absence sociomatrices.

	Density	Av. Path length	Reciprocity	Out-degree centralization	Out-closeness centralization
**Fighting**	0.311	5.432	0.146	0.752	0.717
**Chasing**	0.530	1.545	0.514	0.512	0.376
**Sniffing**	0.826	1.174	0.862	0.190	0.164
**Grooming**	0.364	1.773	0.500	0.198	0.137

Significantly, the fighting network has very high out-degree and out-closeness network centralization scores of >0.7, indicating that this network has an incredibly high concentration of power among few individuals. The chasing network has moderately high network centralizations values of 0.38–0.51 indicating also a concentration of power to relatively few individuals. However, the grooming and sniffing networks have low centralization scores of <0.2 indicating that there is no power structure in these networks. Congruently, both the fighting (p = 0.000) and chasing (p = 0.011) networks had a maximum out-degree that was significantly greater than expected compared to 5,000 randomly generated networks of the exact same size and density. Interestingly, we note that the sniffing network had a trend (p = 0.064) to have a minimum in-degree lower than expected compared to 5,000 random graphs.

#### ii) Comparing individuals across networks

To assess whether the patterning of individual network positions were similar or different across different behavioral networks QAP correlation tests were performed. Each test used 5,000 permutations to obtain a bootstrapped Pearson correlation coefficient and associated p-value (**S3 Table**). A significant correlation was found between the fighting and chasing binary networks suggesting that an individual position in each network was associated with each other (r = 0.402, p = 0.002) but no relationship was found between the fighting and grooming or sniffing networks. An individual's associations in the chasing network was also positively correlated with the sniffing network (r = .328, p < .001). The grooming network was not significantly correlated with any other network.

To examine whether the strength of existing associations between any two individuals is predictive of the strength of the same association in another network, QAP correlation tests were also performed on the fighting, chasing, sniffing and grooming frequency sociomatrices (**S3 Table**). The strength of ties in the fighting network was significantly correlated with the strength of ties in the chasing (r = 0.639, p < .001) and sniffing networks (r = .280 p = .001) but not the gooming network. The strength of existing associations in the chasing, sniffing and grooming networks were all positively correlated with each other (see **S3 Table**), indicating that those individuals who directed these behaviors more frequently at other individuals were also more likely to perform each of the other behaviors to those individuals more frequently than to other individuals with whom they had links.

Following [72] we also compared individual node-level network measures to assess how the patterning of individual network position correlated across networks using Spearman rank correlation tests (a Bonferonni adjusted p-value of 0.0167 was used as each network was compared to 3 others, e.g. fighting with chasing, sniffing, and grooming, the adjusted p-value for significance testing was 0.05/3 = 0.0167). Two clear associations were detected when examining the association between individual network metrics for different behaviors (**S4 Table**). Very strong relationships were found between individual out-degree (rho = 0.78, p<0.005) and out-closeness (rho = 0.67, p = 0.0181) scores in the fighting and chasing networks and between individual in-degree (rho = 0.87, p<0.001) and in-closeness (rho = 0.82, p = 0.001) in the sniffing and chasing networks. Thus individuals that fight many other individuals also chase many other individuals and animals that get chased more also get sniffed more. All other associations did not reach Bonferonni adjusted p-significance criteria, including all associations between individuals' betweenness across networks. Interestingly, no individual network scores of individuals in the grooming network significantly correlated with any other behavioral network.

#### iii) Assessment of individuals' dominance within networks

Individual power scores are shown in **Table 5**. An individual’s Bonacich's power centrality score (rho = 0.71, p<0.01) and hub score in the fighting and chasing networks are significantly associated with one another (rho = 0.71, p<0.01). For all three networks (fighting, chasing, all aggression), individual Bonacich power centrality and hub centrality scores are very tightly correlated indicating that both are reliable measures of power dominance (rhos 0.90–0.99, all p < .0001).

**Table 5:** Individual differences in a) Bonacich’s Power Centrality and b) Kleinberg’s Hub Centrality.

	Fighting BP	Chasing BP	All Aggression BP	Fighting Hub	Chasing Hub	All Aggression Hub
**ev**	0.272	0.201	0.195	1.000	0.937	0.971
**gm**	0.272	0.222	0.202	1.000	1.000	1.000
**ak**	0.132	0.164	0.170	0.553	0.759	0.857
**dw**	0.122	0.046	0.088	0.420	0.232	0.444
**kz**	0.031	0.049	0.046	0.118	0.229	0.247
**gv**	0.019	0.024	0.023	0.078	0.134	0.128
**ip**	0.000	0.025	0.023	0.000	0.094	0.118
**gx**	0.030	0.154	0.106	0.078	0.756	0.564
**iz**	0.122	0.022	0.063	0.470	0.111	0.331
**ps**	0.000	0.022	0.021	0.000	0.098	0.101
**mp**	0.000	0.022	0.021	0.000	0.098	0.101
**oz**	0.000	0.049	0.042	0.000	0.250	0.216

(Calculated from fighting, chasing and all aggression win/loss sociomatrices.)

To examine the relationship between individual differences in dominance and network power measurements, we correlated (using Spearman tests) each individual's I&SI, David's score and Glicko ranking (**Table 2**) against their Bonacich's power and hub score ranking (**Table 5**). Even correcting for multiple comparisons, all fighting dominance rankings were highly correlated with fighting network power rankings (rho ranges 0.85–0.95, all p < .001) and the same relationships were also observed for measures of chasing (rho ranges 0.68–0.94, all p < .02). There is therefore a very high correspondence between traditional metrics of dominance and network power rankings (see **S6 Fig**).

### 3. Neurobiology of individual differences in social dominance and network position

**Fig 5:**
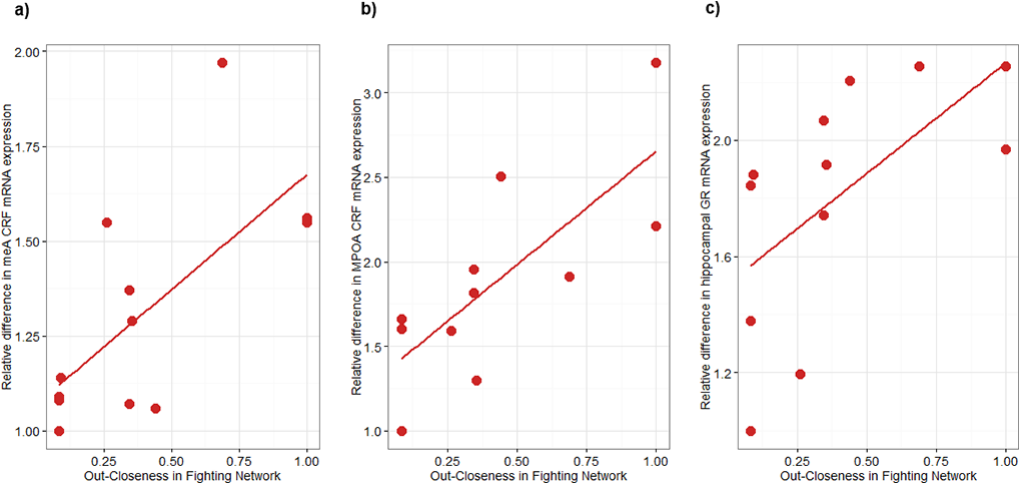
Association between individual differences in Out-Closeness Centrality in the fighting network and relative gene expression. a) CRF mRNA in the Medial Amygdala, b) CRF mRNA in the mPOA, c) GR mRNA in the hippocampus.

The relationship between the level CRF mRNA in the medial amygdala, central amygdala, mPOA and hippocampus, BDNF and GR mRNA expression in the hippocampus and various measures of fighting dominance rank were examined. The PCAs conducted on both the fighting and chasing variables found that component 1 (‘dominance’) accounted for 87% of total variance. All variables loaded onto component one with eigenvalues >0.9 with the exception of in-closeness that loaded with an eigenvalue of -0.77. We therefore also correlated individual component scores against each gene. Results of multiple spearman's rank correlation tests can be found in **S5 Table**. As there is a high degree of inter-correlation between the dominance measures we present all results with individual rho and p-values. In the amygdala, we found very highly significant correlations between level of CRF mRNA in the medial and central nuclei and fighting dominance with more highly ranked individuals having relatively higher levels of CRF mRNA (**S5 Table**). The strongest of these observed relationships were associations with social network measures of power (**Fig 5**). Interestingly, although the associations are in the same direction, medial amygdala and hypothalamic CRF expression was only significantly associated with dominance in the chasing network on a few ranking metrics. No significant relationship was found between fighting dominance and relative hippocampal CRF mRNA. However, relative hippocampal GR and BDNF expression is significantly higher in more dominant individuals of both the fighting and chasing networks. Further, sniffing and grooming network measures of out-Closeness and in-Closeness do not relate to any measure of gene expression in any brain region (**S5 Table.**).

## Discussion

Here we show that by acquiring longitudinal observational data on a group of laboratory mice living in a large, complex environment, that it is possible to acquire a detailed understanding of the social behavior of individuals, the temporal patterning of dyadic social relationships and the overall group social structure. This is achieved through applying statistical methodologies that are routine in field work in primatology and behavioral ecology, and by also using recently developed statistical techniques such as social network analysis and the Glicko pairwise comparison model. In particular, we demonstrate that inbred CD1 male mice have a highly ordered social structure with individual mice having non-random, directionally consistent, social relationships. We demonstrate that all individuals recognize their niche in the social group and are capable of behaving appropriately towards other individuals.

### Mice form a stable dominance hierarchy

All individual mice were observed to be aggressive at least once and to receive aggression at least once, but there was a great deal of inter-individual variability. The directionality of these aggressive behaviors were not random and we found strong evidence for a linear dominance hierarchy when considering fighting behavior, chasing behavior or both aggressive behaviors combined (**Table 1**). Applying De Vries’ method for calculating dominance linearity to frequency interaction sociomatrices, all modified Landau values (h’) were above 0.62, with all p-values being <0.001 indicating strong linearity. We also found that dominance hierarchies based on fighting and chasing had significant steepness, again demonstrating that the agonistic relationships of these mice were organized. Directional consistency was also very high, with 71% of chasing and 93% of fighting interactions being in the direction of dominant to subordinate individuals. This is suggestive that the fighting behavior exhibited by male mice is a stronger enforcer of dominance relationships than chasing behavior, which may occur in the direction of subordinate to dominant.

Our findings were strongly supported by evaluation of global metrics of social networks based upon binarized sociomatrices of each behavior (**Table 4**). The fighting network had extremely low density and reciprocity, and very high average path lengths and out-degree and out-closeness centralization coefficients. Essentially, this describes a social network that has low cohesion, connectedness and compactness, with mostly unidirectional ties between individuals (meaning that fighting behaviors are typically directed), with power and influence being centralized to relatively few individuals. The chasing network showed a similar pattern to the fighting network but not quite as extreme. For instance, the reciprocity and average path length of the chasing network was very similar to the grooming network, but the chasing network did possess moderately high out-degree and out-closeness centralization coefficients indicative of power being restricted to relatively few individuals (though more individuals than the fighting network). Consistent with these findings, we also demonstrated that both the fighting and chasing networks possessed individuals whose maximum out-degree (the highest number of individuals in the group that any one individual attacked or chased) was significantly higher than would be expected for networks of their given size and density, further demonstrating the hierarchical nature of these mice agonistic interactions.

Given the organized hierarchical structure of the fighting and chasing dominance data, we were also able to successfully rate and rank individuals and found a high degree in stability of rank order of individuals over time (**Table 2**). We also introduce a novel method of evaluating the relative rating and rank of an individual’s dominance over time using the Glicko rating system [56]. When considering the final Glicko ratings and deviations (**Fig 3**), it appears that there are broadly two categories of dominant and two categories of subordinate mice. The two most dominant mice have the highest ratings followed by two sub-dominant individuals. Two individuals are the most subordinate with the remaining mice having intermediate rankings. This is somewhat similar to findings of hybrid F1-C57129S male mice housed in groups of four who can be classified as dominant, active subordinate, passive subordinate, or submissive based on agonistic interactions [73].

We also introduce two individual level social network metrics that can be utilized to determine the power of an individual. In our group of mice, we found a statistically very strong relationship between Bonacich’s Power Centrality (which considers individuals who direct behavior towards others who do have relatively fewer connections to be more powerful) and Kleinberg’s Hub Centrality (which considers individuals who have many outgoing ties that connect to individuals with many incoming ties as being more powerful) [64,65]. We also show that these scores were highly correlated with more traditional methodologies of ranking individuals such as I&SI and David Score’s as well as Glicko rating for agonistic behaviors (**Table 3**). We suggest that these network metrics could potentially be very useful additional tools in the study of individual variation of social dominance, as well as in aiding in identifying the social roles of individuals, which is a key aim of network analysis [74,75].

Importantly, it appears that all male mice in this large social group are capable of determining their social rank and behaving appropriately (more aggressively towards subordinates, and more defensively towards dominants). Our findings contrast somewhat with the few previous attempts at investigating dominance structures in mice that typically report that there exists one despotic dominant mouse in a social group with all other individuals being subordinate. There are several reasons for this. Firstly, the ancestral species of mice have extremely flexible social systems meaning that under varied ecological and social conditions that different hierarchical styles of social groups may emerge from despotic to completely linear [5,71,76–78]. In the laboratory, differences in housing conditions or experimental design may change social structure. For instance, despotic dominance appears to be more common among small groups of fewer than 5 or 6 individuals living in standard sized cages [9,79–82], whereas if mice are given more space, it has been reported that groups will start to form some form of social hierarchy with previously subordinate animals starting to dominate some other individuals [10,83]. Differences may of course be also due to strain differences in aggressive and subordinate behaviors, which are known to exist [9,84], though we purposefully chose CD1 mice due to their common use in social behavior tests and willingness to engage in agonistic interactions [41,85].

### Relationship between agonistic and other social behaviors

We also examined at the individual, relationship and group levels how the patterning of agonistic behaviors related to the spatial patterning of two other social behaviors–sniffing and allogrooming.

#### Sniffing

In many studies of dyadic interactions, the time spent socially investigating (sniffing) another animal is most often utilized as a proxy for the ‘sociability’ of that animal [1]. Our data suggest that in a large group of male mice, sniffing between individuals occurs very frequently and appears to be related to chasing behavior. The high levels of mutual sniffing interactions and interconnectivity of the sniffing network was evidenced by the high density, reciprocity and low average path length of the network (**Table 4**). Nevertheless, the network density was still significantly less than a theoretical density of 1 indicating the presence of highly differentiated social relationships and the reciprocity was not higher than expected by chance for a network of its given size and density (**S2 Table**). Several findings indicate that the organization of sniffing within the social group was somewhat related to patterns of social dominance. Firstly, the sniffing network had a minimum in-degree of 5, which was lower than expected compared to random graphs of the same size and density (p = 0.06). This suggests that the most dominant individuals are getting approached and sniffed less than would be expected by chance. Secondly, using QAP correlations, both the presence/absence and the strength of a network tie were highly positively associated between the chasing and sniffing networks and the strength of associations between the fighting and sniffing network were also associated (**S3 Table**). Finally, we also found that the individual in-degree and in-closeness network scores were highly correlated between chasing and sniffing networks, indicating that animals who were chased by many different individuals are also those who are likely to get sniffed by many different social partners (**S4 Table**).

Significantly, even though chasing and fighting networks are closely related on multiple measures, there was no association in terms of the presence of ties between the sniffing and fighting networks. This is again suggestive of subtle differences between fighting and chasing in how dominance is exerted in the social group. From this data, we would concluded that social investigation in the form of sniffing may actually be a dominance related behavior with subordinate mice avoiding sniffing dominant males, at least when occurring between males who are familiar to one another in a stable social group, in contrast to how it is often used to determine the general ‘sociability’ of mice. Some support for this comes from studies of scent marking in mice, as subordinate mice are known to avoid investigating the urine scent marks of more dominant individuals [86,87].

### Allogrooming

Allogrooming between mice is commonly assumed in studies of dyadic and small group social interactions of mice to serve a general affiliative prosocial function as is common in large mammals [88–90]. However, it is not clear that this is unequivocally the case in laboratory mice and this behavior may actually serve multiple functions (including agonisitic, affiliative or neutral) dependent upon the relative social status of the animals and other social contextual factors [84,91]. Our data indicate that allogrooming is largely structurally separate from all other behaviors considered (fighting, chasing, sniffing). QAP correlation tests revealed that the presence/absence of a tie between two individuals in the grooming network was not predictive of that tie being present or absent in any other behavioral network (**S2 Table**). Grooming network metrics at the individual level were also not correlated with the same individual’s network metrics in other behavioral networks (**S4 Table**). This does not necessarily imply that this behavior is therefore affiliative by default. Our finding of a correlation between the strength of existing associations in the grooming network with the chasing and sniffing networks (though not the fighting network) is suggestive that grooming interactions may not solely be affiliative. Nevertheless, we also found that the grooming network’s density was very low indicating that social relationships for this behavior are highly specific (**Table 4**). We also found a trend for the reciprocity of grooming ties to be higher than would be expected by chance, suggesting that perhaps this behavior may serve some mutually affiliative function between pairs of mice. One other study that examined the direction of allogrooming interactions in laboratory mice reported that for various inbred strains the most common grooming interactions were between subordinates, followed by subordinates grooming dominants and lastly dominants grooming other dominants [9]. One interpretation of our and these data may be that allogrooming most often provides social buffering and support between reciprocally subordinate mice, and when occurring reciprocally between dominant and subordinate mice may serve to maintain the dominance hierarchy but have opposite functions (to maintain dominance and to ameliorate aggression respectively).

### Dominance rank and brain gene expression

We found a very strong relationship between relative individual mRNA levels of CRF in the medial amygdala, central amygdala and MPOA with fighting dominance rank and network measures of power and out-closeness (**S5 Table** and **Fig 5**). More dominant individuals have relatively higher mRNA expression of CRF. Medial amygdala and hypothalamic CRF mRNA expression was also associated with dominance and power in the chasing network but less strongly than the fighting network. No relationship between hippocampal CRF and fighting or chasing dominance or network measures of power were found. These relationships appear to be specific to dominance as no relationship was observed between CRF expression and grooming or sniffing network metrics.

There is strong evidence for the coordinated involvement of the MeA, CeA and mPOA in the regulation of social behaviors relevant to social dominance [21,25,27]. The MeA, which receives inputs from the accessory olfactory system and main olfactory system via the cortical nucleus of the amygdala, projects to multiple brain regions implicated in the regulation of social behavior [92]. The mPOA and other medial hypothalamic nuclei receive direct innervations from the anteroventral and posterodorsal divisions of the MeA with relevance for the modulation of defensive and aggressive behavior. Both the MeA and mPOA show increases in cFos expression in both aggressive and subordinate individuals following dyadic resident-intruder encounters [35,93]. Though the exact role of the mPOA during these agonistic social encounters remains to be elucidated, it is thought to modulate an individual’s arousal in response to socially relevant cues [35]. Further, the medial and capsular subdivisions of the CeA, a nucleus that is critical for the coordination of sensory experience, social learning and memory and emotional behaviors [29], receives substantial innervations from the MeA [94]. Descending projections from the medial division of the CeA innervate the hypothalamus providing an additional association between the MeA and hypothalamus [95]. Unsurprisingly, lesions of the MeA lead to multiple deficits in social memory as well as the expression of and learning about aggressive and subordinate behaviors demonstrating the key importance of this nucleus as a critical regulator of social dominance and subordination behaviors [31,96,97]. More recently, discrete glutametergic and GABAergic neuronal populations in the MeA have been reported to regulate social aggression [27].

A role for CRF in maintaining social hierarchies in other species such as teleost fish has already been established [98,99]. In mammals, although it has not been explicitly examined whether CRF regulates social hierarchy formation and maintenance, CRF does influence many important features of those social behaviors required to function efficiently in a social hierarchy. For instance, it has been shown that ICV CRF administration in rats [100] and over-expressing CRF in neurons in mice [101] leads to increased social investigation, improved social memory and recognition. Intra-amygdalar administration of CRF in rats increases both aggression and social investigation [37], whilst intra-amygdalar administration with CRF-R1 antagonists inhibits the induction of submissive behaviors in mice when given immediately after social defeat stress [102]. Indeed, there is a lot of support for the role of CRF being an important facilitator for the formation of emotional memories particularly those related to social avoidance [103–105]. We suggest that our findings may support a hypothesis that those individuals with higher CRF expression in the amygdala (MeA and CeA) as well as the mPOA achieve higher dominance rank through CRF’s actions, elevating levels of aggression and improving the abilities of mice to detect and remember social chemosensory information such that they are more likely to exhibit appropriate subordinate or dominant behavior towards other individuals. Determining if this may occur through the direct actions of CRF on CRF1R receptors or the CRF-binding-protein (CRFBP) which occur at high levels in the amygdala and hypothalamus [106,107], or though the interaction of CRF expressing neurons with other neuroendocrine systems such as serotonergic, dopaminergic or noradrenergic neurons [39] will form the basis of future work.

We found that the relative hippocampal expression of BDNF mRNA was significantly related to all metrics of dominance rank and individual network power with more dominant animals having higher BDNF expression (**S5 Table**). The strong association between BDNF and chasing dominance may be related to increased territoriality of more dominant mice. More dominant male mice living in large groups are known to increase their activity levels compared to prior to group formation [81] and exhibit territorial patrolling behavior [10]. A consistent finding is that physical activity increases the expression of hippocampal BDNF, neural plasticity and cognition [108]. We hypothesize that the increased physical activity by more dominant chasing animals may have driven the observed BDNF expression differences. Potentially, the neural plasticity induced by increased territorial dominance may promote the required neural plasticity and improved spatial memory required of patrolling dominant animals. Interestingly, one recent study reports that hippocampal neurogenesis was related to the territoriality and roaming entropy of mice living in large groups [11]. Several studies also indicate that hippocampal BDNF may promote learning about an individual’s social role following aggressive encounters [41,109], particularly in more dominant individuals [43]. Taken together with our data, it could be hypothesized that individual mice who are able to ascend a dominance hierarchy do so in part because of their elevated hippocampal BDNF levels.

It is also possible that individual differences in hippocampal BDNF expression may be related to variations in social stress experienced, as it is well known that mice that experience social defeat or other forms of social stress show reductions in hippocampal BDNF levels [110,111]. However, this does not explain why the observed relationship between BDNF and chasing dominance was higher than that between fighting dominance. Further, the relationship between individual BDNF expression levels and in- and out-closeness were equivalent. If the differences in BDNF expression were specifically related to differences in social stress experienced, it would be expected that in-closeness would have a stronger relationship to gene expression levels than out-closeness.

Relative hippocampal GR mRNA levels were strongly associated with dominance rank, with more dominant individuals having higher expression and those individuals who receive a lot of aggression from multiple individuals having lower levels of GR expression. Although it should not be assumed that more subordinate animals in a social group are necessarily those animals are the most socially stressed as particular social, environmental and demographic characteristics of a group could mean that dominants or even middle-ranking individuals may be the most socially stressed individuals [112,113], our finding is congruent with a vast literature detailing that individuals who experience social stress possess fewer GRs in the hippocampus due to down-regulation of gene expression. Consistent with this, others have reported that dominant mice tend to have lower baseline CORT levels than subordinates amongst those living in small groups (fewer than 5) [114] and an attenuated stress response in response to physical stressors [115], though others do also report that these baseline differences may disappear in mice that have lived together for periods of several weeks [116], and may even be higher in dominant mice living in enriched conditions [117].

## Conclusions

The study of laboratory mice social behavior has proliferated recently due to the increased desire to develop animal models of human mental disease [1,19]. Most of our understanding of the neurobiology of social behavior however still relies on dyadic social interactions. We and others [11–14] have proposed that there is a growing need to embrace and explore the complexity social behavior at individual, relationships and group levels in more ecologically and ethologically relevant paradigms. Here, we have shown that we are able to study the dominance interactions of male mice using traditional and novel statistical approaches including social network analysis. We have demonstrated that large groups of male mice form fighting and chasing dominance hierarchies with individual mice having a definitive rank order, and that individual differences in rank and measures of power are associated with changes in MeA, CeA and mPOA CRF mRNA expression and hippocampal BDNF and GR expression. Although we are unable in the current study to determine whether these individual differences in gene expression are causally responsible for the divergence in social ranks or are a consequence of these changes, we aim to evaluate this in future work by determining if it is possible to alter dominance network position and structure via targeting gene expression of individual mice in a brain-region specific manner.

## Supporting Information

S1 Fig The distribution of final Glicko ratings +/- deviations of each of 12 mice as calculated using a range of constant values (cvals) of 1 to 20. (TIF)

S2 Fig Presence/absence sociomatrices. a) Fighting, b) Chasing, c) All aggression, d) Sniffing, e) Grooming. A ‘1’ indicates that individuals in rows directed that behavior to individuals in columns at least once during the observation period. A ‘0’ indicates that that behavior was never observed to have occurred directed from individuals in rows to individuals in columns.
(TIF)

S3 Fig Win/loss sociomatrices. a) Fighting, b) Chasing, c) All aggression. A ‘1’ indicates that individuals in rows directed each particular behavior more frequently to individuals in columns than they received the same behavior from individuals in columns.
(TIF)

S4 Fig Results of Permutation Test to test for significance of linearity. The observed Landau’s modified h’ value of dominance hierarchy linearity for each agonistic sociomatrix (fighting, chasing, all aggression) is compared against the values obtained after 10,000 randomizations of each sociomatrix. The dashed line represents the observed h’ value. (*** p<0.001).
(TIF)

S5 Fig High individual consistency in dominance rank based on Glicko ratings for fighting data using a range of c-values (1–20). (TIF)

S6 Fig Consistency of Dominance Methods. Relationship between Kleinberg’s Hub Centrality calculated from the fighting win/loss sociomatrix and a) I&SI rank, b) David’s Scores, c) Final Glicko Rating.
(TIF)

S1 Supplementary Codes and Data –All raw data and R scripts used in the data analysis.
(ZIP)

S1 Table The ethogram used for behavioral observations. (DOCX)

S2 Table Densities of individual behavior networks based on presence/absence sociomatrices. All networks are significantly different from possessing a density of 1 indicating specificity of social ties in the network.
(DOCX)

S3 Table Pearson correlations between individual behavior networks calculated using QAP. For a) presence/absence of a tie, b) strength of a tie. (*** p<0.001, ** p<0.01, * p<0.05, ^$^p < .1).
(DOCX)

S4 Table Individual differences in individual level network metrics calculated from fighting, chasing, sniffing and grooming presence/absence sociomatrices. (DOCX)

S5 Table Associations between relative gene expression and dominance measures. (DOCX)
