# Supplementary figures and images for: A Social Network Approach Reveals Associations between Mouse Social Dominance and Brain Gene Expression
Source: PLoS One. 2015 Jul 30;10(7):e0134509. doi: 10.1371/journal.pone.0134509 (PMC4520683; doi:10.1371/journal.pone.0134509)

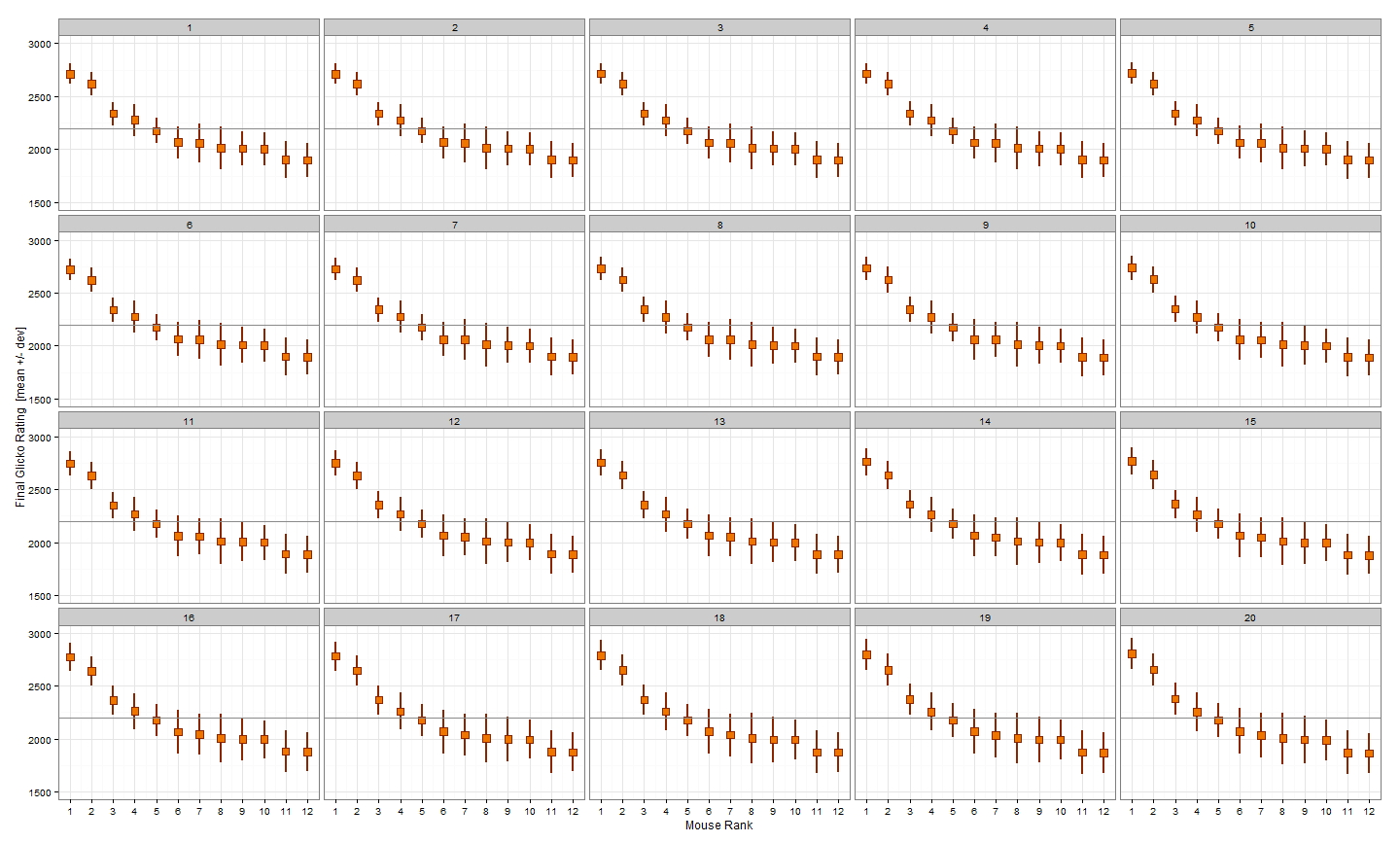

Supplement: S1 Fig — (TIF) [file pone.0134509.s001.tif]

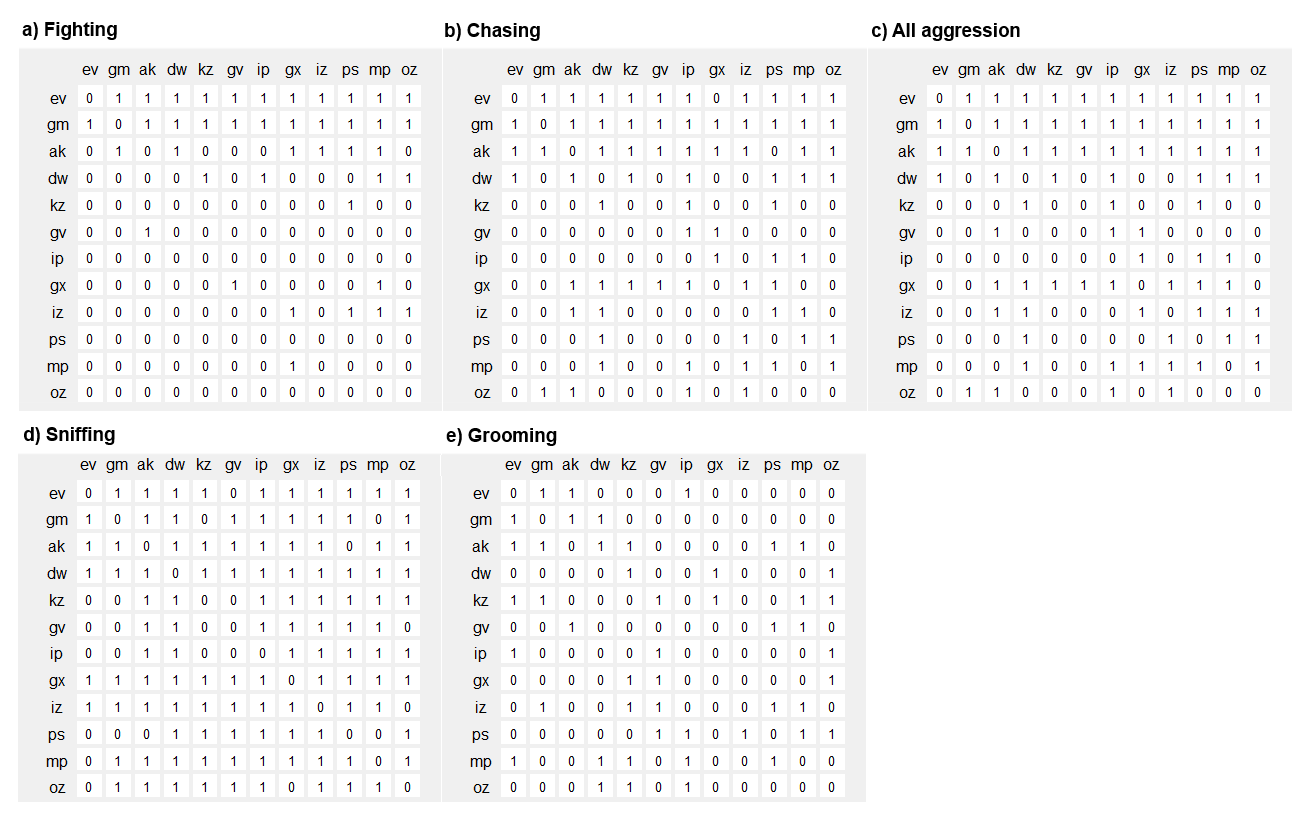

Supplement: S2 Fig — a) Fighting, b) Chasing, c) All aggression, d) Sniffing, e) Grooming. A ‘1’ indicates that individuals in rows directed that behavior to individuals in columns at least once during the observation period. A ‘0’ indicates that that behavior was never observed to have occurred directed from individuals in rows to individuals in columns. (TIF) [file pone.0134509.s002.tif]

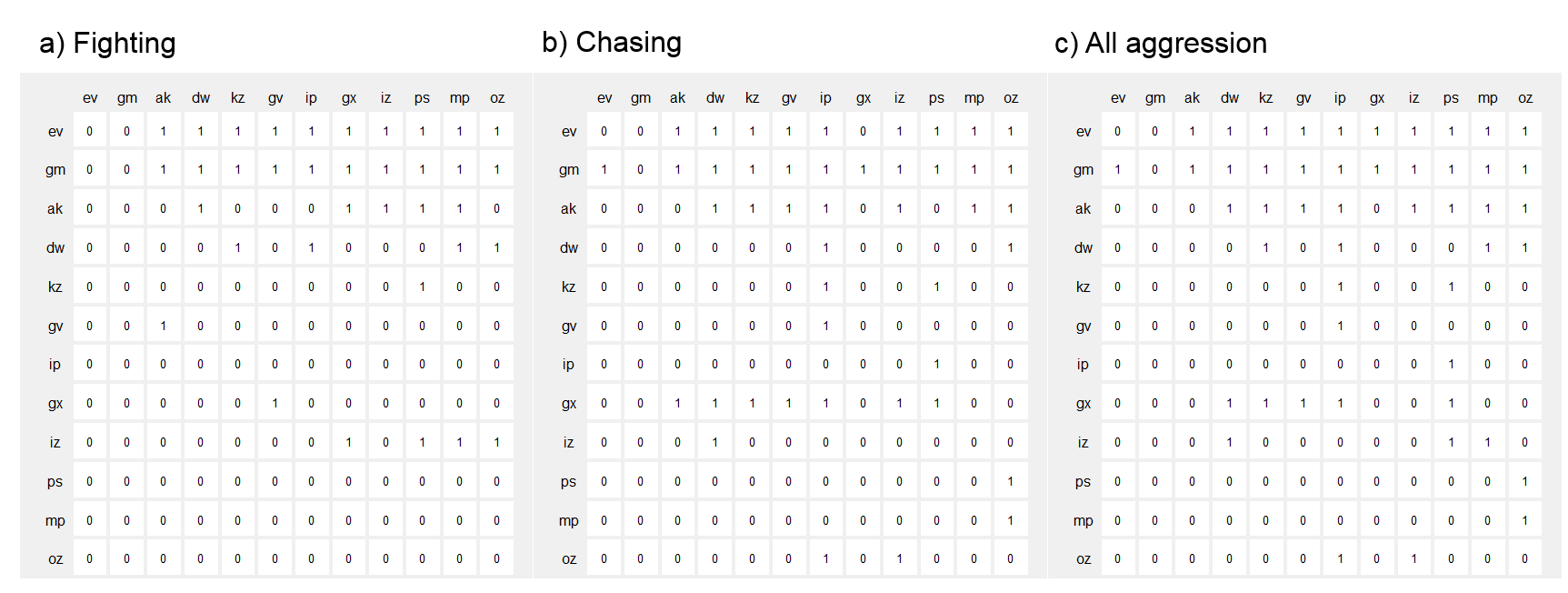

Supplement: S3 Fig — a) Fighting, b) Chasing, c) All aggression. A ‘1’ indicates that individuals in rows directed each particular behavior more frequently to individuals in columns than they received the same behavior from individuals in columns. (TIF) [file pone.0134509.s003.tif]

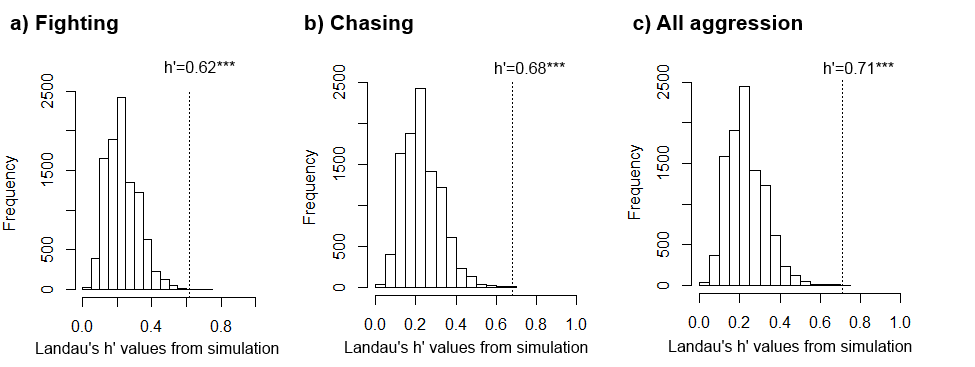

Supplement: S4 Fig — The observed Landau’s modified h’ value of dominance hierarchy linearity for each agonistic sociomatrix (fighting, chasing, all aggression) is compared against the values obtained after 10,000 randomizations of each sociomatrix. The dashed line represents the observed h’ value. (*** p<0.001). (TIF) [file pone.0134509.s004.tif]

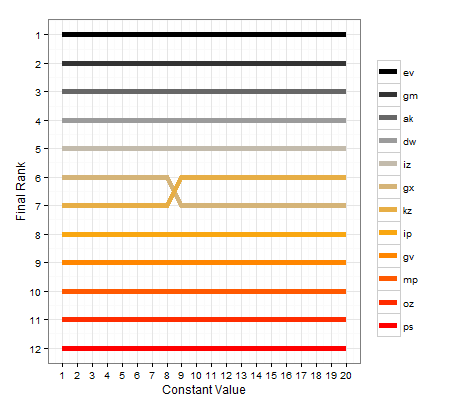

Supplement: S5 Fig — (TIF) [file pone.0134509.s005.tif]

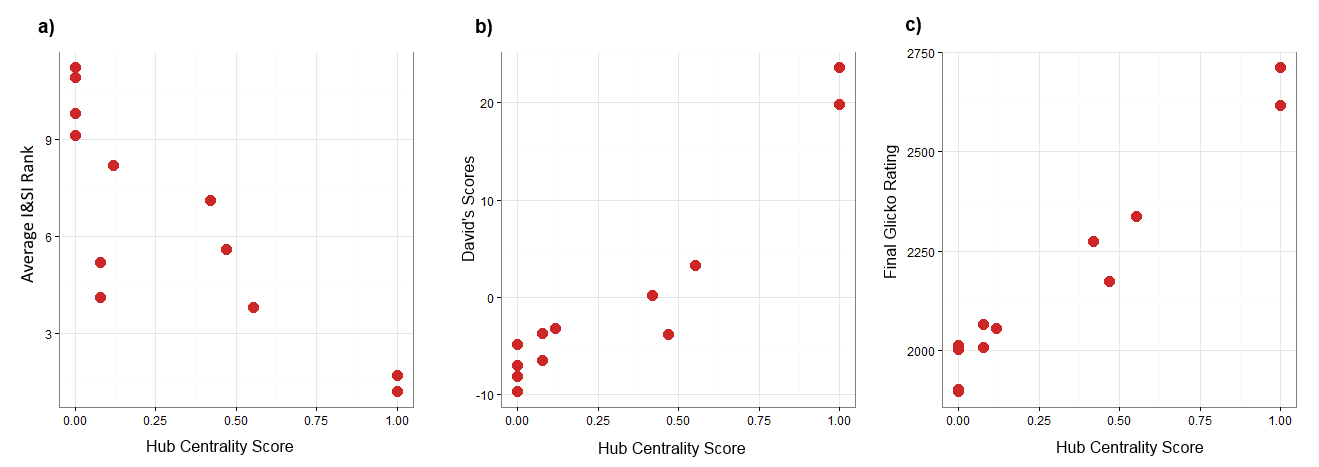

Supplement: S6 Fig — Relationship between Kleinberg’s Hub Centrality calculated from the fighting win/loss sociomatrix and a) I&SI rank, b) David’s Scores, c) Final Glicko Rating. (TIF) [file pone.0134509.s006.tif]
