# Supplementary material for: A Social Network Approach Reveals Associations between Mouse Social Dominance and Brain Gene Expression
Source: PLoS One. 2015 Jul 30;10(7):e0134509. doi: 10.1371/journal.pone.0134509 (PMC4520683; doi:10.1371/journal.pone.0134509)
Supplement: S1 Table — (DOCX) [file pone.0134509.s008.docx]

**S1 Table.** The ethogram used for behavioral observations.

| **Behavior** | **Description** |
| --- | --- |
| Fighting | The focal individual lunges or bites the target individual for at least 2 seconds. |
| Chasing | The focal individual follows the target individual rapidly and aggressively whilst the target individual attempts to flee. |
| Sniffing | The focal individual approaches and makes an olfactory investigation of the head, body or anogential region of the target individual for at least 2 seconds. |
| Allogrooming | The focal individual grooms with their paws and mouth the fur and/or face of the target individual. |
| Self-grooming | The focal individual grooms themselves with their paws. |
| Huddling | The focal individual is resting in direct body contact with at least one other individual. |
| Rest alone | The focal individual is resting by themselves without body contact to any other individual. |
| Eating | The focal individual is eating |
| Drinking | The focal individual is drinking |
