# Supplementary material for: A Social Network Approach Reveals Associations between Mouse Social Dominance and Brain Gene Expression
Source: PLoS One. 2015 Jul 30;10(7):e0134509. doi: 10.1371/journal.pone.0134509 (PMC4520683; doi:10.1371/journal.pone.0134509)
Supplement: S2 Table — All networks are significantly different from possessing a density of 1 indicating specificity of social ties in the network. (DOCX) [file pone.0134509.s009.docx]

**S2 Table.** Densities of individual behavior networks based on presence/absence sociomatrices. All networks are significantly different from possessing a density of 1 indicating specificity of social ties in the network.

|  | **Fighting** | **Chasing** | **Sniffing** | **Grooming** |
| --- | --- | --- | --- | --- |
| **Density** | 0.311 | 0.530 | 0.826 | 0.364 |
| **Z-score** | -8.83 | -6.55 | -2.25 | -9.45 |
| **p-value** | <0.001 | <0.001 | 0.031 | <0.001 |
