# Supplementary material for: A Social Network Approach Reveals Associations between Mouse Social Dominance and Brain Gene Expression
Source: PLoS One. 2015 Jul 30;10(7):e0134509. doi: 10.1371/journal.pone.0134509 (PMC4520683; doi:10.1371/journal.pone.0134509)
Supplement: S3 Table — For a) presence/absence of a tie, b) strength of a tie. (*** p<0.001, ** p<0.01, * p<0.05, $p < .1). (DOCX) [file pone.0134509.s010.docx]

**S3 Table.** Pearson correlations between individual behavior networks calculated using QAP for **a)** presence/absence of a tie, **b)** strength of a tie. (*** p<0.001, ** p<0.01, * p<0.05, ^$^p<.1).

**a)**

|  | **Chasing** | **Sniffing** | **Grooming** |
| --- | --- | --- | --- |
| **Fighting** | 0.402** | 0.093 | 0.037 |
| **Chasing** |  | 0.328*** | -0.046 |
| **Sniffing** |  |  | 0.015 |

**b)**

|  | **Chasing** | **Sniffing** | **Grooming** |
| --- | --- | --- | --- |
| **Fighting** | 0.639*** | 0.280*** | 0.013 |
| **Chasing** |  | 0.494*** | 0.151* |
| **Sniffing** |  |  | 0.281** |
