# Supplementary material for: A Social Network Approach Reveals Associations between Mouse Social Dominance and Brain Gene Expression
Source: PLoS One. 2015 Jul 30;10(7):e0134509. doi: 10.1371/journal.pone.0134509 (PMC4520683; doi:10.1371/journal.pone.0134509)
Supplement: S4 Table — (DOCX) [file pone.0134509.s011.docx]

**S4 Table. Individual differences in individual level network metrics calculated from fighting, chasing, sniffing and grooming presence/absence sociomatrices.**

**a) Out-Degree**

|  | **Fighting** | **Chasing** | **Sniffing** | **Grooming** |
| --- | --- | --- | --- | --- |
| **ev** | 11 | 10 | 10 | 3 |
| **gm** | 11 | 11 | 9 | 3 |
| **ak** | 6 | 10 | 10 | 6 |
| **dw** | 4 | 7 | 11 | 3 |
| **kz** | 1 | 3 | 8 | 6 |
| **gv** | 1 | 2 | 7 | 3 |
| **ip** | 0 | 3 | 7 | 3 |
| **gx** | 2 | 7 | 11 | 3 |
| **iz** | 4 | 4 | 10 | 5 |
| **ps** | 0 | 4 | 7 | 5 |
| **mp** | 1 | 5 | 10 | 5 |
| **oz** | 0 | 4 | 9 | 3 |

**b) In-Degree**

|  | **Fighting** | **Chasing** | **Sniffing** | **Grooming** |
| --- | --- | --- | --- | --- |
| **ev** | 1 | 3 | 5 | 5 |
| **gm** | 2 | 3 | 7 | 4 |
| **ak** | 3 | 6 | 10 | 3 |
| **dw** | 3 | 8 | 11 | 4 |
| **kz** | 3 | 5 | 8 | 6 |
| **gv** | 3 | 4 | 8 | 5 |
| **ip** | 3 | 9 | 11 | 4 |
| **gx** | 5 | 4 | 10 | 2 |
| **iz** | 3 | 7 | 11 | 1 |
| **ps** | 5 | 8 | 10 | 4 |
| **mp** | 6 | 7 | 9 | 5 |
| **oz** | 4 | 6 | 9 | 5 |

**c) Out-Closeness**

|  | **Fighting** | **Chasing** | **Sniffing** | **Grooming** |
| --- | --- | --- | --- | --- |
| **ev** | 1.000 | 0.917 | 0.917 | 0.524 |
| **gm** | 1.000 | 1.000 | 0.846 | 0.524 |
| **ak** | 0.688 | 0.917 | 0.917 | 0.688 |
| **dw** | 0.355 | 0.733 | 1.000 | 0.478 |
| **kz** | 0.091 | 0.524 | 0.786 | 0.647 |
| **gv** | 0.440 | 0.478 | 0.733 | 0.550 |
| **ip** | 0.083 | 0.524 | 0.733 | 0.524 |
| **gx** | 0.344 | 0.733 | 1.000 | 0.550 |
| **iz** | 0.344 | 0.611 | 0.917 | 0.647 |
| **ps** | 0.083 | 0.550 | 0.733 | 0.611 |
| **mp** | 0.262 | 0.611 | 0.917 | 0.647 |
| **oz** | 0.083 | 0.611 | 0.846 | 0.478 |

**d) In-Closeness**

|  | **Fighting** | **Chasing** | **Sniffing** | **Grooming** |
| --- | --- | --- | --- | --- |
| **ev** | 0.149 | 0.524 | 0.647 | 0.647 |
| **gm** | 0.162 | 0.500 | 0.733 | 0.611 |
| **ak** | 0.175 | 0.688 | 0.912 | 0.524 |
| **dw** | 0.172 | 0.786 | 1.000 | 0.611 |
| **kz** | 0.190 | 0.647 | 0.786 | 0.688 |
| **gv** | 0.183 | 0.524 | 0.786 | 0.611 |
| **ip** | 0.190 | 0.846 | 1.000 | 0.611 |
| **gx** | 0.193 | 0.579 | 0.917 | 0.458 |
| **iz** | 0.169 | 0.733 | 1.000 | 0.367 |
| **ps** | 0.275 | 0.786 | 0.917 | 0.550 |
| **mp** | 0.196 | 0.733 | 0.846 | 0.647 |
| **oz** | 0.204 | 0.647 | 0.846 | 0.647 |

**e) Betweenness Centrality**

|  | **Fighting** | **Chasing** | **Sniffing** | **Grooming** |
| --- | --- | --- | --- | --- |
| **ev** | 0.000 | 0.019 | 0.004 | 0.053 |
| **gm** | 0.117 | 0.033 | 0.008 | 0.030 |
| **ak** | 0.309 | 0.138 | 0.283 | 0.110 |
| **dw** | 0.058 | 0.131 | 0.037 | 0.064 |
| **kz** | 0.009 | 0.001 | 0.006 | 0.188 |
| **gv** | 0.255 | 0.002 | 0.006 | 0.122 |
| **ip** | 0.000 | 0.065 | 0.008 | 0.049 |
| **gx** | 0.205 | 0.111 | 0.034 | 0.015 |
| **iz** | 0.012 | 0.022 | 0.035 | 0.014 |
| **ps** | 0.000 | 0.047 | 0.011 | 0.131 |
| **mp** | 0.055 | 0.030 | 0.016 | 0.081 |
| **oz** | 0.000 | 0.053 | 0.016 | 0.072 |
