# Supplementary material for: A Social Network Approach Reveals Associations between Mouse Social Dominance and Brain Gene Expression
Source: PLoS One. 2015 Jul 30;10(7):e0134509. doi: 10.1371/journal.pone.0134509 (PMC4520683; doi:10.1371/journal.pone.0134509)
Supplement: S5 Table — (DOCX) [file pone.0134509.s012.docx]

**S5 Table. Associations between relative gene expression and dominance measures.**

**a) Fighting**

|  | **MeA CRF** | **CeA CRF** | **mPOA CRF** | **Hipp CRF** | **Hipp BDNF** | **Hipp GR** |
| --- | --- | --- | --- | --- | --- | --- |
| **I&SI Rank** | -0.46 | -0.71* | -0.87*** | -0.07 | -0.66* | -0.84*** |
| **Glicko rating** | 0.62* | 0.57^$^ | 0.56^$^ | 0.07 | 0.59* | 0.69* |
| **David’s scores** | 0.50^$^ | 0.63^*^ | 0.63* | 0.07 | 0.40 | 0.76** |
| **Bonacich’s Power** | 0.68* | 0.67* | 0.65* | 0.02 | 0.62* | 0.69* |
| **Hub Score** | 0.73** | 0.66* | 0.65* | 0.01 | 0.60* | 0.65* |
| **Out Closeness** | 0.61* | 0.73** | 0.74** | 0.21 | 0.66* | 0.81** |
| **In Closeness** | -0.53^$^ | -0.74** | -0.58^$^ | -0.09 | -0.50^$^ | -0.59* |
| **Component1 - PCA** | 0.60* | 0.72* | 0.63*  + | 0.15 | 0.63* | 0.78** |

**b) Chasing**

|  | **MeA CRF** | **CeA CRF** | **mPOA CRF** | **Hipp CRF** | **Hipp BDNF** | **Hipp GR** |
| --- | --- | --- | --- | --- | --- | --- |
| **I&SI Rank** | -.056^$^ | -0.45 | -0.68* | -0.13 | -0.59* | -0.72* |
| **Glicko rating** | 0.49 | 0.28 | 0.50 | -0.06 | 0.63* | 0.71* |
| **David’s scores** | 0.51^$^ | 0.51^$^ | 0.68* | 0.02 | 0.58^$^ | 0.86*** |
| **Bonacich’s Power** | 0.38 | 0.24 | 0.50 | -0.07 | 0.47 | 0.75** |
| **Hub Score** | 0.55^$^ | 0.34 | 0.52 | 0.09 | 0.66* | 0.79** |
| **Out Closeness** | 0.74* | 0.17 | 0.31 | 0.11 | 0.70* | 0.49 |
| **In Closeness** | -0.22 | -0.43 | -0.72* | 0.04 | -0.48 | -0.67* |
| **Component1 - PCA** | 0.51^$^ | 0.42  42 | 0.65* | 0.06  0.5700 | 0.57^$^ | 0.83** |

**c) Sniffing and Grooming**

|  | **MeA CRF** | **CeA CRF** | **mPOA CRF** | **Hipp CRF** | **Hipp BDNF** | **Hipp GR** |
| --- | --- | --- | --- | --- | --- | --- |
| **sniffing (Out Closeness)** | 0.50^$^ | 0.17 | -0.11 | 0.29 | 0.53^$^ | 0.34 |
| **sniffing (In Closeness)** | -0.29 | -0.14 | -0.50 | -0.06 | -0.15 | -0.40 |
| **grooming (Out Closeness)** | 0.29 | 0.22 | 0.18 | 0.16 | 0.14 | -0.04 |
| **grooming (In Closeness)** | 0.09 | -0.26 | -0.23 | 0.24 | -0.42 | -0.08 |

(*** p <.001, ** p<.01, *p<.05, ^$^p<.1).
